# Course of Cholera

**Published:** 1849-03

**Authors:** 


*Course of Cholera—its rate of progress—its mortality—its preliminary stage.*—One of the most remarkable facts connected with the Asiatic Cholera is, that, in its present progress throughout Europe, it should follow so nearly the course which it took in 1830–1. The researches of Dr. Lasègue have shown that this analogy not only exists in respect to the time at which the places are visited, but in respect to the duration of the disease at each place. The cholera appeared at Tiflis on the 5th May, 1830; at Astrachan on the 21st June; and, ascending the Volga, it reached the Russian province of Kasan on the 17th of September in the same year. In 1847 the cholera made its appearance at Tiflis on the 1st June; at Astrachan on the 31st July; and reached Kasan on the 4th October. In 1830, as in 1847, it took five months to traverse the same district.

In 1830, taking the course of the Dnieper, it reached Stavropol on the 6th September; Novo Tscherkosk on the 10th; Taganrog on the 8th October, and Kiev on the 8th January, 1831. In 1847, the cholera broke out at Stavropol on the 16th July; at Novo Tscherkosk on the 30th; at Taganrog on the 15th August; and at Kiev on the 5th October. Although, as a general rule, those districts, towns, and cities which were visited in 1830, have been attacked by the disease on the present occasion, Dr. Lasègue has pointed out one very remarkable exception. In 1830–'1, the disease spread through the provinces on the western frontiers of Russia; but n 1847, from some singular and unexplained cause, these provinces have escaped; and to this may perhaps be ascribed our immunity from the disease up to the present time.

The ravages of the disease were suspended in the winter of 1830, as well as in that of 1847. In both instances Moscow formed the extreme western limit of the pestilence; and in the spring of 1831, as well as in that of 1848, the disease resumed its course. It appeared in St. Petersburgh on the 25th June, 1831, and it broke out in this city, and spread through it with fearful rapidity, on the 16th June, 1848. It attacked Berlin on the 31st August, 1831, and on the 15th August, 1848. It is well known that the disease first appeared in England, at Sunderland, on the 26th October, 1831; and it will be a remarkable confirmation of the analogies hitherto observed in its progress on the continent, if the rumor that it has now appeared in one of our seaports on the northeastern coast should prove to be well founded. If we are to be guided by this analogy, the cholera may not appear in the metropolis until the ensuing winter. The first cases were announced in London on the 13th February, 1832, and they occurred in the immediate vicinity of the docks. The disease appeared in Paris in the spring of 1832, and that city, therefore, may escape the visitation until the spring of 1849.

It is worthy of remark that in 1830–'1, as in 1847–'8, the cholera has manifested itself chiefly in the great lines of intercourse along freqented roads, and the banks of navigable rivers, attacking chiefly towns and cities where the population was most dense, producing the largest amount of mortality in its first onset, then slowly diminishing in severity, and finally disappearing to reappear in a neighboring locality. According to Dr. Lasègue, the greatest rapidity with which the cholera has spread over any locality has not exceeded a rate of from 250 to 300 miles a month. This comparatively slow progress, together with its advance in the face of prevailing winds, is very unlike the usual mode of diffusion of a purely epidemic disease.

It was confidently announced a year since, that the cholera, as it then prevailed on the continent, had lost much of its severity, and was far less mortal than the disease of 1830–'1. This statement, however, is contrary to fact. In comparing its fatality in the countries to which its ravages have been hitherto confined, the deaths are, even comparatively speaking, more numerous than on the former visitation. In the Russian empire alone, between the months of April and August, 1848, no less than 505,328 persons were attacked with cholera, and of these 210,836 died—a mortality of more than *forty per cent.* The tables published by the Sanitary Board of St. Petersburgh show, that in estimating the mortality produced by the disease in fourteen of the principal cities of the empire, it appears, that in 1847, of 21,295 attacked, 11,361 died; and in 1830-'1, of 15,559 attacked, 9,018 died. The proportion of those attacked to the total population, was about the same. Thus, in the Russian empire, the proportion of deaths to the attacks was—

| | |
|---|---|
| In 1830-'1 | In 1847 |
| 1 to 1·7 | 1 to 1·8 |

and the proportion of those attacked to the total population was—

| | |
|---|---|
| In 1830-'1 | In 1847 |
| 1 to 19·6 inhabitants. | 1 to 19·7 ditto. |

Even in Berlin, where it was alleged that cholera had appeared in a much milder form, in the present invasion, we find that from the 15th August to the 1st of September, the attacks were 377, and the deaths 235—or no less than 64 per cent! This great mortality may be ascribed to the severely epidemic form in which the disease has prevailed in that city.

Experience has added one fact of importance in a prophylactic view to our knowledge of this terrible pestilence. As a general rule, the Russian practitioners have observed, that the suddenness of an attack of cholera is apparent, and not real—it is in its severe form, the secondary and intractable stage of a disease which, at its commencement, is comparatively mild and tractable; and which, if taken in time may be without difficulty arrested by simple remedies. Their experience has led them to the conclusion, that diarrhea is a precursory symptom of an attack of Asiatic cholera; and this diarrhea may or may not be attended with pain in the abdomen.—There is very frequently an entire absence of pain—a circumstance which leads to the neglect of means for remedying what appears to be a temporary disorder, but which may turn out to be the forerunner of the fatal malady. In the diarrhea preceding cholera, when pain has been noticed, it has been simple uneasiness, with a sense of contraction in the bowels. The number of evacuations may be from one to six or more daily: they retain in this stage their fecal color and odor, and are in this respect very different from those alvine discharges, which occur in the more advanced stage of the disorder; for these have no fecal odor or color, and resemble rice water. This simple diarrhea may, therefore, be considered to be the commencement of an attack of Asiatic cholera, this name being applied only to the last stage of the disease.

The diarrheal stage may last only a few hours—two or three days, or even longer. If properly treated, the second stage may be entirely averted—if neglected, this will commence suddenly and violently with those severe symptoms which are commonly the precursors of death. The suddenness of an attack of cholera is, therefore, only apparent—when inquiry has been made, the milder stage although in some instances of very short duration, had really existed, but was overlooked. These views of the Russian physicians are strongly confirmed by the observations made by Dr. Monneret, the French Medical Commissioner at Constantinople and Trebizond. We cannot now enter into the question, whether cholera does or does not in some instances destroy life without a diarrheal stage. This is quite foreign to our object, which is that of endeavoring to find out some warning symptom of the disease, so that the person attacked may be placed on his guard, and induced to seek medical advice without loss of time. Let us admit, for the sake of argument, that of 100 cases diarrhea may not appear in 14; our remarks are directed to the 86 who suffer from this very common premonitory symptom.

It follows, from the preceding observations, when cholera is prevalent in a locality, the slightest disturbance of the bowels requires attention. Considering the possible risk incurred by neglect, the fact that there is only one evacuation more than common, or that the evacuation is more liquid than natural, should receive immediate notice. If the diarrhea really depend on other causes, and not on cholera, no mischief will follow from its arrest by medicine;—if, however, it depend on the cholera-poison beginning already to operate on the body—then, by resorting to treatment, a life may be saved. It must be remembered that we have no means of determining *á priori* on what the diarrhea depends; and, contrary to popular belief, it appears that the diarrhea of cholera is really of a more mild description than that which arises from any local cause of irritation in the bowels.—*London Med. Gaz.*—*Am. Jour. Med. Sci.*

